# Fatal *S. aureus* Hemorrhagic Pneumonia: Genetic Analysis of a Unique Clinical Isolate Producing both PVL and TSST-1

**DOI:** 10.1371/journal.pone.0027246

**Published:** 2011-11-03

**Authors:** Zhi Li, Dennis L. Stevens, Stephanie M. Hamilton, Tanyalak Parimon, Yongsheng Ma, Angela M. Kearns, Richard W. Ellis, Amy E. Bryant

**Affiliations:** 1 Veterans Affairs Medical Center, Boise, Idaho, United States of America; 2 University of Washington School of Medicine, Seattle, Washington, United States of America; 3 Idaho State University, Pocatello, Idaho, United States of America; 4 Microbiology Services Division, Health Protection Agency, London, United Kingdom; 5 Microbiology Department, South Tyneside NHS Foundation Trust, South Shields, United Kingdom; Baylor College of Medicine, United States of America

## Abstract

In 2008, an unusual strain of methicillin-sensitive *Staphylococcus aureus* (MSSA68111), producing both Panton-Valentine leukocidin (PVL) and toxic shock syndrome toxin-1 (TSST-1), was isolated from a fatal case of necrotizing pneumonia. Because PVL/TSST-1 co-production in *S. aureus* is rare, we characterized the molecular organization of these toxin genes in strain 68111. MSSA68111 carries the PVL genes within a novel temperate prophage we call ФPVLv68111 that is most similar, though not identical, to phage ФPVL – a phage type that is relatively rare worldwide. The TSST-1 gene (*tst*) in MSSA68111 is carried on a unique staphylococcal pathogenicity island (SaPI) we call SaPI68111. Features of SaPI68111 suggest it likely arose through multiple major recombination events with other known SaPIs. Both ФPVLv68111 and SaPI68111 are fully mobilizable and therefore transmissible to other strains. Taken together, these findings suggest that hypervirulent *S. aureus* have the potential to emerge worldwide.

## Introduction

*Staphylococcus aureus* infections range in severity from superficial abscesses to complicated deep soft tissue infections and toxic shock syndrome (TSS) [1], [2]. The pathogenesis of these diverse infections is attributed to multiple extracellular toxins including the Panton-Valentine leukocidin (PVL) and Toxic Shock Syndrome Toxin 1 (TSST-1). PVL has been epidemiologically linked to severe community-associated MRSA (CA-MRSA) infections [3], [4] while TSST-1 clearly mediates shock and organ failure in staphylococcal TSS [5], [6].

Historically, a single strain of *S. aureus* rarely produced both PVL and TSST-1. However, in 2005, one British report documented the TSST-1 gene in 4 of 30 PVL-positive isolates [7], one of which was associated with severe pneumonia [7]. Subsequently, twenty *S. aureus* isolates (15 MSSA; 5 MRSA) harboring both PVL and TSST-1 toxin genes were reported in the United Kingdom [8]. Of these, seventeen strains were from one of three clonal complex (CC) lineages: twelve (60%) belonged to lineage CC30 and five were either CC5 or CC22. The other three strains could not be assigned to any known clonal complex. Four of the 5 MRSA strains (80%) were multi-drug resistant. Eight of these isolates (40%) were associated with serious diseases including pneumonia, empyema, deep-seated abscesses and toxic shock. Nine patients presented with abscess or other skin infections [9]. One fatal case of necrotizing pneumonia was also reported in a 14-year-old child who presented initially with sore throat and pyrexia, and then deteriorated rapidly, developing hypotension and multiple organ failure [9].

In this report, we describe the genetic makeup of the hypervirulent TSST-1/PVL co-producing MSSA isolated from this fatal case (MSSA68111; CC30) [9]. Our results demonstrate that MSSA68111 produces both PVL and TSST-1 toxins. Further, its PVL-carrying phage and TSST-1-carrying pathogenicity island (SaPI) are both unique and not heretofore reported in *S. aureus*. Both novel genetic elements are fully mobilizable, suggesting that the emergence and dissemination of hypervirulent *S. aureus* may be forthcoming worldwide.

## Materials and Methods

### *S. aureus*

MSSA68111 was from a fatal case of necrotizing pneumonia in a 14-year-old child who presented initially with sore throat and pyrexia, and then deteriorated rapidly, developing hypotension and multiple organ failure [9]. It is penicillin-resistant but sensitive to erythromycin, tetracycline, vancomycin and linezolid [8]. It is of the MLST-CC30 lineage (sequence type ST776, a triple locus variant of ST30;*spa* type t399), harbors *agr* type 3 and is gene-positive for *sec* but negative for *sea, seb, seg, seh* and *sei* [8] (and unpublished data). TSST-1-positive MSSA strains were reported as being highly associated with the CC30 lineage, which is a very common genotype worldwide and is the most predominant lineage reported in the United Kingdom, Ireland, and Switzerland [10]–[13]. Laboratory strain ATCC 49775 (American Type Culture Collection) harbors the PVL genes (herein referred to as *lukF*-PV and *lukS*-PV) and produces *LukF*/S-PV [14], [15]. A clinical CA-MRSA isolate, strain 04-014, produces TSST-1 but not PVL [16]. Two clinical USA300 MRSA strains, 934814 and 09-301-02119, were from patients with septic arthritis and fatal post-influenza pneumonia, respectively. MSSA laboratory strain RN4220 is a restriction-deficient derivative of the 8325-4 strain [17]. MRSA strain MW2 (USA400) was originally isolated from a 16-month-old girl with fatal septicaemia [18].

*S. aureus* were routinely cultured in Mueller-Hinton II broth; no differences in growth rates were observed. For analysis of toxin production, washed *S. aureus* from overnight cultures were diluted to 1-3×10^6^ CFU/mL in fresh media and grown at 37°C in 5% CO_2_ with shaking (200 rpm) for 20 h (yields 1-4×10^9^ CFU/mL). Culture supernatant fluids were filter sterilized and frozen at −70°C until assayed for toxins.

### Toxin assays

TSST-1 and PVL were measured by ELISA [16], [19]. Alpha-hemolysin activity was measured by lysis of rabbit erythrocytes [19]. All assays were run in duplicate or triplicate and included 2*–*3 biological replicates.

### PVL induction and Phage DNA Purification

Exponential phase *S. aureus* (OD_600_ = 0.8) in CYPG (Casamino acids 10 g; yeast extract, 10 g; NaCl, 5 g;0.5% glucose and 0.06 M phosphoglycerate in 1 L) were treated with 1 µg/ml mitomycin C and cultured for 3.5 hrs at 32°C with slow shaking (80 rpm). The culture was centrifuged (8,000Xg, 20 min, 4°C) and the filtered (0.22 um) supernatant was treated with DNase I (0.5 U/ml) and RNase A (0.25 mg/ml) at 37°C for 1 hr. After centrifugation (20,000Xg, 1 hr, 4°C), the pellet was resuspended in TES Buffer (100 mM Tris pH 8, 100 mM EDTA, 1% SDS) in the presence of proteinase K (1 mg/ml). After 30 min at 55°C, the DNA was extracted twice with phenol-chloroform and precipitated with two volumes of cold 100% ethanol. The DNA pellet was washed twice with 70% ethanol and resuspended in 100 ul H_2_O. The junction sequence of induced PVL phage in MSSA68111 was PCR-amplified using an inverse primer set targeting the integrase (*int*) (PVL-*int:* AGCAATTATGAAAAGGGT-AGGACA) and PVL (*lukF*-PV: AGTTGATTGGGAAAATCATACAGTT) genes.

### Southern Blot Analysis of PVL Phage DNA

Restriction enzyme digestion of PVL phage DNA and Southern blot analysis was performed using standard methods. Specific DNA fragments were detected using a chemiluminescence system (North2South, Thermo Fisher, Rockford, IL). *lukS*-PV probe was PCR-amplified using the primers *lukS*-left: TGTATCTCCTGAGCCTTTTTCA and *lukS*-right: CAGACAATGAATTACCCCCATT.

### Chromosomal Localization of PVL Phage

Bacteria were grown in CYPG to OD_625_ = 0.6. Genomic DNA was extracted with a commercial kit (Qiagen). The chromosomal insertion site of PVL phage was PCR-verified with the primer pair phiPVL-reverse (to the *lukF*-PV gene: AGTTGATTGGGAAAATCATACAGTT) and phiPVL-forward (to a conserved hypothetical protein: TCCATCGTTTGAATTGCTTG).

### Tst-SaPI induction and Genomic DNA Purification

Bacteria were grown in CYPG to OD_625_ = 0.15, centrifuged, resuspended in CYPG-Phage Buffer (1∶1) at a density of OD_625_ = 0.05 and infected with phage 11 or phage 80α at a multiplicity of 6∶1 or 3∶1. Cultures were grown at 32°C with slow shaking (80 rpm) for 1 hr. Samples (8 ml) were centrifuged and the bacterial pellet resuspended in 100 ul GET buffer (0.1 M glucose, 0.01 M EDTA and 0.05 M Tris-HCl, pH 8) and lysed with lysostaphin (0.5 mg/ml) in the presence of RNaseA (0.5 mg/ml) at 37°C for 1 hr. The lysate was further digested with proteinase K (0.5 mg/ml) at 55°C for 3 hrs. Genomic DNA was then extracted with a commercial kit (Qiagen). The junction sequence of induced SaPI68111 was PCR-amplified using an inverse primer set targeting the *sel* (CTGGACCTACATCGCCGTTA) and SaPI-integrase (GTGTTGGATGAGCAATTACCAA) genes. The chromosomal insertion site of SaPI68111 was further PCR-verified with the primer pair *ssrP*-up:TAGCGGAAAATCGTAAAGCAAG and integrase-down:GTGTTGGATGAGCAAT-TACCAA.

### Quantitative Real-Time PCR

DNase-treated total RNA (1 µg) was reverse transcribed using M-MuLV enzyme (New England Biolab, Ipswich, MA) and random hexamer primers and dNTPs (Invitrogen, Carlsbad, CA). cDNA was diluted fifty-fold in nuclease-free water and used for real-time PCR performed with a 7500 Fast Real-time PCR System (Applied Biosystems, Carlsbad, CA) using the RT^2^ real-time SYBR green/Rox PCR master mix (SuperArray, Frederick, MD) and primer sets: (1) LukS-PV-up:TGTATCTCCTGAGCCTTTTTCA and LukS-PV-down: CAGACAATGAATTACCCCCATT (Accession: YP_494079); (2) RecA-up:TGCAGAACA-GCTTAAACAAGGA and RecA-down:ACTCTAAATGGTGGTGCCACTT (Accession: L25893); (3) gyrase-up:ATGTAGCAAGCCTTCCAGGTAA and gyrase-down:CAGAGTCCCCTT-CGACTAAGAA (Accession: YP_492727); (4) TSST-1-up:TCGCTACAGATTTTACCCCTGT and TSST-1-down:CGTTTGTAGATGCTTTTGCAGT (Accession: NC_002745). Relative gene expression was determined using the 2^-ÄÄCt^ method [20]. The fold change in expression of the gene of interest was normalized to the internal control gene (*Gyrase* B) and made relative to the calibrator sample (Time = 0).

## Results

### 1. Toxin Production

In our initial studies, we compared by ELISA the levels of PVL and TSST-1 in 20-hr culture supernatant from *S. aureus* strain 68111 with known PVL- (ATCC49775 and a clinical MRSA isolate 934814) or TSST-1-producing (04-014) isolates (see details in Materials and Methods section) [14*]*–[**16]. Our analysis showed that MSSA68111 produces 160.24 ng/mL PVL in 20-hr culture, which is significantly lower than that from the well-characterized PVL-producing MSSA strain ATCC49775 (Table 1), but comparable to that obtained from a clinical strain 934814 (USA300) (Table 1). Similarly, the production of TSST-1 in MSSA68111 is also relatively low, yielding 716.5 ng/mL, whereas the TSST-1 positive clinical isolate, strain 04-014, had a strongly positive ELISA result, producing 6739 ng/mL (Table 2).

**Table 1:** PVL in 20-hr bacterial culture supernatants.

Strain	Concentration of LukF-PV (ng/mL)±Std Dev
ATCC 49775	852.7±207.2
934814 (USA300)	113.74±15.4
68111	160.24±28.8

**Table 2:** TSST-1 in 20-hr bacterial culture supernatants.

Strain	Concentration of TSST-1 (ng/mL) ±Std Dev
04*–*014	6739±533.2
68111	716.5±14.8

**Figure 1:**
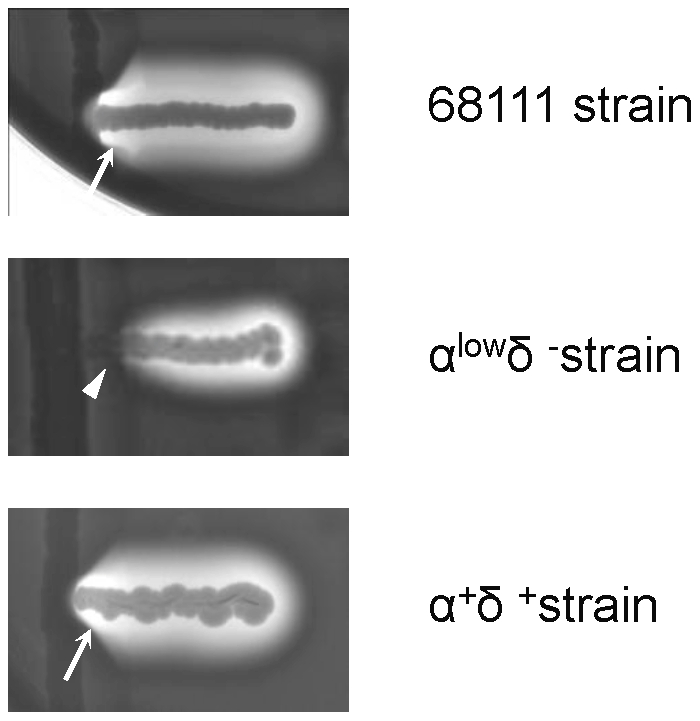
Alpha- and delta-hemolysin activities of *S. aureus* strain 68111. A plate-based screening method that capitalizes on beta-hemolysin-mediated effects on alpha- and delta-hemolysin activities was used to examine extracellular production of these toxins in MSSA68111. For this, beta-hemolysin-producing *S. aureus* strain RN4220 was streaked vertically on sheep blood agar. MSSA68111 (top) and two laboratory control strains (α-hemolysin^low^/δ-hemolysin**^-^** [middle] and α-hemolysin^+^/δ-hemolysin**^+^** [bottom]) were then streaked perpendicularly to RN4220. Hemolysis was assessed after 24 hrs incubation at 37°C (no cold shock applied). At the junction between the test strains and RN4220, alpha-hemolysin activity is inhibited (arrowhead); the presence of delta-hemolysin is observed as an enhanced area of hemolysis (arrows).

Further, various hemolytic activities of MSSA68111 were assessed. When 20-hr culture supernatant of MSSA68111 was assayed for alpha-toxin with rabbit erythrocytes, a titer of 246 HU/ml was obtained (Table 3). With the same supernatant, no “hot-cold” hemolysis could be detected on sheep erythrocytes, indicating that staphylococcal beta-toxin is either absent or present only in a small amount in this strain (data not shown). Last, for the delta-hemolysin, we adapted a quick-screening methodology established by Herbert [21] and Traber [22] which takes advantage of the fact that delta-hemolysin activity is enhanced by beta-hemolysin. Thus, by streaking test strains perpendicularly to strain RN4220, which produces only beta-hemolysin, one can visually identify delta-hemolysin production in test strains as an enhanced area of hemolysis where the two strains come in near to one another. As shown in Figure 1, MSSA68111 produces a significant amount of delta-hemolysin, indicated by the increased area of hemolytic activity when it nears strain RN4220. This same assay also can be used to detect alpha-hemolysin production in test strains since alpha-hemolysin activity is inhibited by beta-hemolysin. Using this technique, alpha-hemolysin production by MSSA68111 was also demonstrated (Figure 1). The moderate alpha- and delta-hemolysin activities, as well as production of TSST-1 (albeit in low amounts) suggested MSSA68111 carries an intact Agr/RNAIII operon.

**Table:** **Table 3.** alpha-hemolysin in 20-hr bacterial culture supernatants.

Strain	Alpha Hemolytic Activity (HU/mL)
09*–*301*–*02119 (USA300)	310
ATCC 49775	98
68111	246

Having confirmed that MSSA68111 does indeed produce both TSST-1 and PVL – a phenomenon that is rare among *S. aureus* strains - we investigated the genetic organization of these genes in this unique clinical isolate.

### 2. Characterization of PVL-Carrying Phage in MSSA68111

#### 2.1. PVL phage lineage and mobility

The genes for PVL are commonly carried by one of six lysogenic bacteriophages [18], [23*]*–[**27] having two distinct phage-head morphologies: icosahedral (ФPVL and Ф108PVL) or elongated (ФSLT, ФSa2 mw, ФSa2958 and ФSa2usa) [18], [23*]*–[**27]. Ma *et al.* recently reported a 2-step PCR-based scheme for characterizing *S. aureus* PVL-encoding phages [28]. The first round of PCR identifies the morphological group using primers specific to the gene lineage between *lukS*-PV and the tail gene. The second round of PCR establishes the phage type using primers recognizing phage-specific structures. With this approach, we determined that MSSA68111 carries an icosahedral-head type PVL-encoding phage, because PCR-1 (amplifying phage portal and tail genes) and PCR-3 (amplifying *lukS*-PV to group-specific tail gene) were positive, whereas PCRs-2 and -4 (amplifying genes specific for elongated-head type phage) were negative. Further, our PCR-5 result (targeting to phage specific structures) showed a PCR product of 1411 bps suggesting that the PVL-carrying phage in MSSA68111 belongs to the ФPVL, but not Ф108 PVL, lineage.

**Figure 2:**
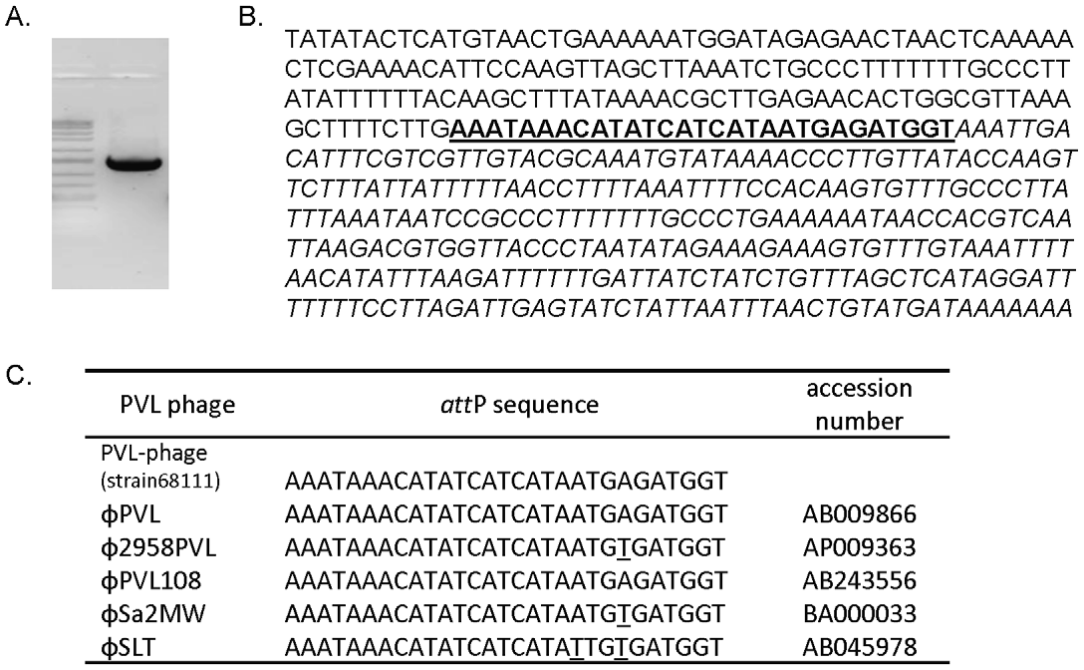
DNA sequence of the *attP* junction of MSSA68111 PVL phage. (**A.**) PCR was performed with an inverse primer set using mitomycin C-elicited phage genomic DNA from MSSA68111 as a template. PCR products were separated by agarose gel electrophoresis and stained with ethidium bromide. (**B.**) Nucleotide sequence of the 68111 PCR product was determined. The sequence originating from PVL phage integrase is printed in upper-case letters, and that derived from *lukF*-PV gene is shown in italic letters, and twenty-nine base pairs of the core *att*P sequence are bolded and underlined. (**C.**) This *att*P sequence of MSSA68111 PVL phage was compared to that from other published PVL-carrying phages. Nucleotide variations were underlined.

To assess the mobility of this PVL phage, we treated MSSA68111 with mitomycin C since it induces the lytic cycle of many temperate phages. After 2*–*4 hrs, the culture visibly cleared, suggestive of phage-meditated bacterial lysis. To confirm this, phage DNA was purified and amplified with an inverse primer set which targets the integrase and *lukF*-PV genes. The resultant PCR product (Figure 2A) suggested that the PVL-carrying phage in MSSA68111 did indeed become lytic following mitomycin C treatment. Commercial sequencing of the PCR product with subsequent GenBank BLAST searching demonstrated that the *attP* junction sequence of MSSA68111's PVL-carrying phage was similar to that in other known PVL-phage lineages (Figures 2B, 2C), demonstrating that the nucleotide sequences of the integrase as well as the chromosomal integration site are highly conserved among different PVL-carrying phages.

**Figure 3:**
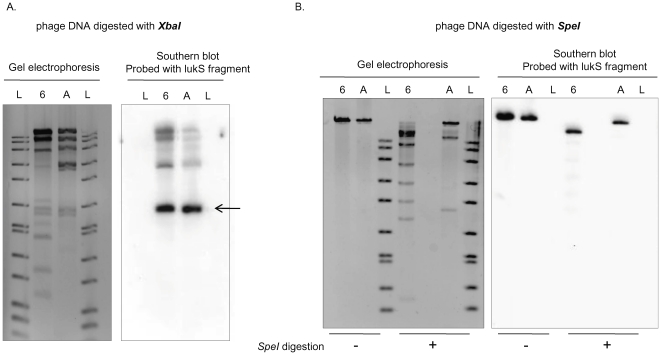
Southern blot analysis of PVL phage genomic DNA from strain 68111 and strain ATCC49775. Phage DNA from MSSA68111 (Lanes designated “6”) and from strain ATCC49775 (Lanes “A”) was digested with either *XbaI* (**A.**) or *SpeI* (**B.**). In both **A** and **B**, the panels on the left are photographs of ethidium bromide-stained gels; the panels on the right are Southern blots hybridized with the *lukS*-PV probe. 1 kb DNA ladders (Lanes “L”) are shown. The *lukS*-PV probe hybridized to identical 2 kb fragments in *XbaI* -digested phage DNA from both strains **(A,** arrow**)** whereas *SpeI-*digestion produced different sized *lukS*-PV fragments (**B.**).

We next compared the restriction profiles of the mitomycin C-elicited PVL-phage genomic DNA from MSSA68111 and ATCC49775 (carries ФPVL [25]) by Southern blot analysis using a *lukS*-PV-specific probe. Identical hybridization patterns were seen following *XbaI*, but not *SpeI*, digestion (Figures 3A, 3B). Thus, the PVL-carrying bacteriophage in MSSA68111 has a similar, but not identical, restriction profile to ФPVL. We propose the name ФPVLv68111 for this ФPVL variant. Last, sequence analysis of *LukSF*-PV genes in MSSA68111 indicated they belonged to the H1 variant with an H176R amino acid substitution. This information is consistent with the work of O'Hara et al [29], showing that H variants of *LukSF-PV* genes were mostly carried by *mecA* ^-^ *S. aureus* isolates found mainly outside the United States.

#### 2.2. Characterization of ФPVLv68111 integration site

**Figure 4:**
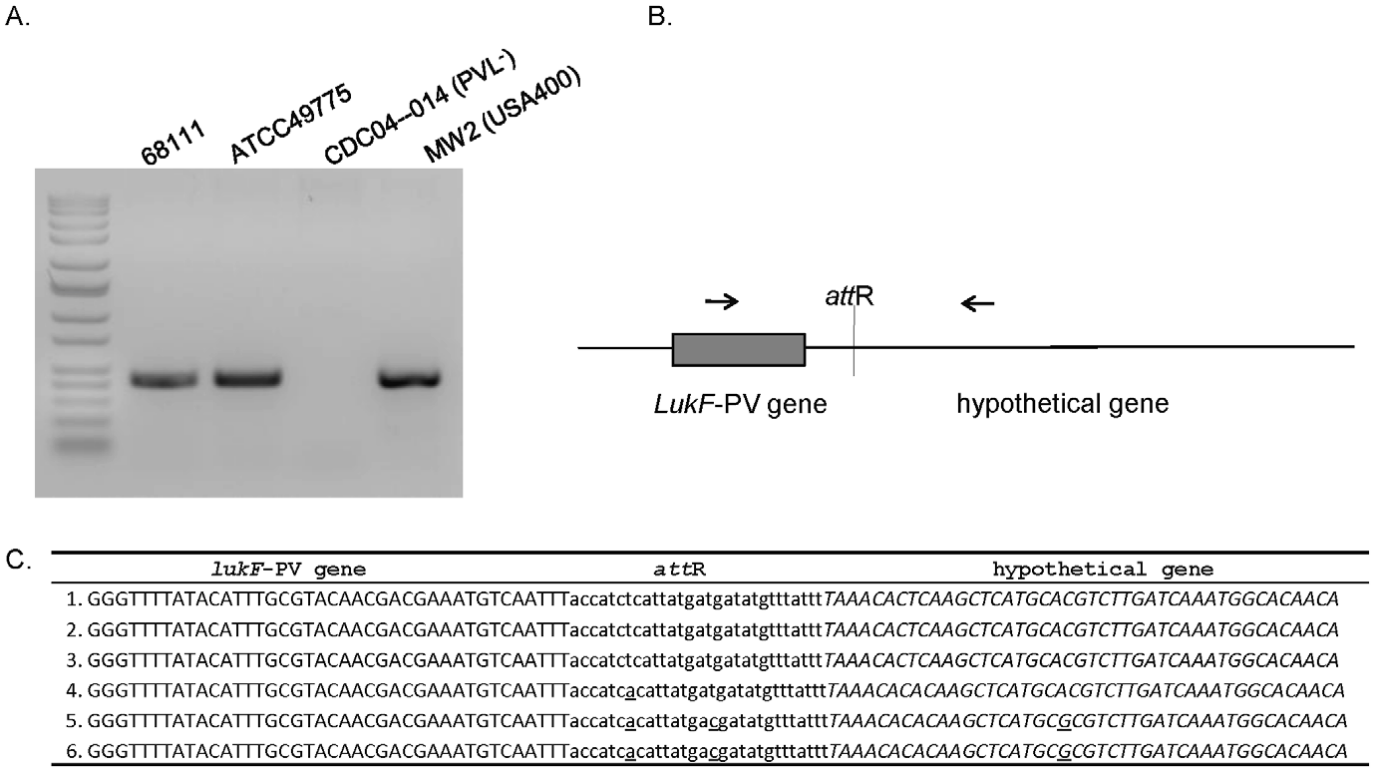
DNA sequences of integration site of PVL phage in bacterial genome. (**A.**) PCR was performed with primers, as shown in (**B.**) using bacterial genomic DNA as templates. PCR products were separated by agarose gel electrophoresis and stained with ethidium bromide. (**C.**) The sequence of PCR product from MSSA68111 was determined and is shown relative to other strains carrying PVL phage. The sequence originating from the phage PVL genomic DNA including *lukF*-PV gene is printed in upper-case letters, and that derived from the bacterial chromosome, a conserved hypothetical protein, is shown in italic letters. Twenty-nine base pairs of core *attR* sequence are bolded and underlined. Nucleotide variations are underlined. 1.ФPVLv68111; 2. ΦPVL (strain ATCC49775 [25]); 3. ΦPVL108 (Accession: AB243556); 4. Φ2958 PVL (Accession:AP009363); 5. ΦSLT (Accession: CP000255); 6. ΦSa2 MW (Accession: BA000033).

To investigate the chromosomal insertion site of ФPVLv68111, we performed PCR based on the known phage PVL insertion sites in the other strains (e.g., ATCC49775, MW2) using primers that hybridize to the *lukF*-PV gene and to a hypothetical gene (Figures 4A, 4B). Commercial sequencing of the resultant PCR product revealed a 29 nucleotide region of the ФPVLv68111 *att*R site (lysogenic form) that was identical to *att*P sequence of ФPVLv68111 (lytic form; Figure 2C), and the position at which the phage inserts into the hypothetical gene of the host chromosome was well conserved among currently known PVL-producing strains (Figure 4C).

### 3. Characterization of Tst-Carrying SaPI in MSSA68111

#### 3.1. Gene organization of *sel-sec-tst*

Several *S. aureus* exotoxin genes cluster within pathogenicity islands (SaPIs) [30], [31]. MSSA68111 was reported to be gene-positive for the exotoxins SEL, SEC and TSST-1 [9] (coded for by *sel, sec* and *tst,* respectively) which are commonly clustered in SaPI1 [32], SaPI2 [30], [33], SaPIn1/m1 [34] or SaPIbov [35], [36]. To investigate the organization of these toxin genes in MSSA68111, PCR was performed using primers based on the known SaPIn1 and SaPIbov sequences. The size of the PCR products and corresponding sequencing data demonstrated that *sel-sec-tst* were clustered sequentially in MSSA68111. Like SaPIn1 or SaPIm1, the *sec* and *tst* genes are approximately 2 kb apart and in opposite orientations (data not shown).

#### 3.2. Mobility of *tst*-carrying SaPI by helper phage

In the excision-replication-packaging (ERP) cycle, temperate bacteriophages can induce excision and replication of SaPIs and can package the ensuing DNA into special small phage-like particles using proteins supplied by the helper phage. Using genomic DNA electrophoresis [32], we assessed two common staphylococcal helper phages, phage 80α or phage 11, for their ability to mobilize the *tst*-carrying SaPI in MSSA68111. We did not observe the typical SaPI-specific band on a stained agarose gel containing bacterial genomic DNA following helper phage induction (data not shown), suggesting that this SaPI did not undergo massive helper phage-mediated ERP.

**Figure 5:**
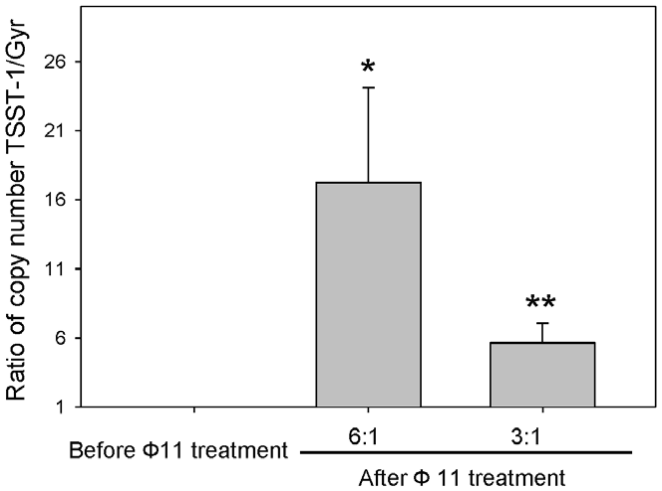
Induction (excision/replication) of *tst*-SaPI elements by phage11. Following superinfection with phage 11 as described in *Materials and Methods*, total genomic DNA was purified and analyzed for *tst* and *gyr* genes by real-time PCR. Relative copy numbers of *tst* versus *gyr* are presented. Data are means±SD (n = 4); p*<0.05; p**<0.01 vs control (before Ф11 treatment) (paired *t*-test).

To increase the sensitivity of detection of ERP, we used real-time qPCR-based methods to quantify the copy number of *tst*-SaPI within MSSA68111 host cells (Figure 5). *Tst* and *gyr* were the target and the reference genes, respectively. Preliminary studies (data not shown) demonstrated that *S. aureus* genome contained only one copy each of *tst* and *gyr* and that the amplification efficiencies of these genes (calculated from the slope of the standard curve generated with serial dilutions of cDNA template for each gene) were equivalent (1.92 vs 1.90, respectively). Thus, the copy ratio of *tst* to *gyr* before SaPI induction was 1. In contrast, following superinfection with phage 11, the *tst* amplification curve was left-shifted indicating increased copy number of the *tst* gene compared to the *gyr* gene. The *tst*-carrying SaPI copy number relative to the reference gene *gyr* could be given by the ratio: [(E*tst*) ^ΔCt*tst*(control-treated)^]/[(E*gyr*) ^ΔCgyr(control-treated)^] [37]. As shown in Figure 5, after 1.5 hrs of exposure to helper phage 11 at MOI of 3∶1 or 6∶1, the relative *tst*-carrying SaPI copy number in MSSA68111 was dose-dependently increased 5.6-fold and 17.2-fold, respectively.

In contrast, no excised form of *tst*-carrying SaPI was found following UV- or mitomycin C treatment or phage 80α induction either by electrophoretic analysis of sheared whole cell lysates or by the above qPCR method (data not shown). We conclude that the excision of *tst*-SaPI in MSSA68111 is highly phage 11-specific.

#### 3.3. Characterization of the integrase of *tst*-carrying SaPI

**Figure 6:**
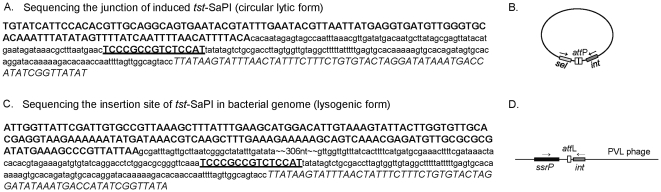
Sequence analyses of *tst*-carrying SaPI in MSSA68111. (**A.**) Junctional sequence of circulated SaPI (lytic form) following helper phage 11 induction. *Sel* coding region is bolded; integrase of *tst*-SaPI is shown in italic letter and 15 base pairs of core *attP* are bolded and underlined. Other intergenic regions are presented in lower-case font. A schematic illustration of the PCR strategy used to amplify this junctional region is shown in (**B.**). (**C.**) Sequence of integration site of *tst*-SaPI (lysogenic form) in bacterial genome. *SsrP* coding region is bolded, integrase of *tst*-SaPI is shown in italic letter and 15 base pairs of core *attL* are bolded and underlined. Other intergenic regions are presented in lower-case font. A schematic illustration of the PCR strategy used to amplify the integration site of *tst*-SaPI is shown in (**D.**).

To determine the integrase sequence and chromosomal integration site of the *tst*-carrying SaPI in MSSA68111, an outward-directed PCR on genomic DNA was conducted following helper phage 11 induction (Figure 6A). Sequencing of the PCR product showed a 15-bp direct repeat occurs at the junction between *sel* and integrase as indicated in Figure 6. The *att* core (5′-TCCCGCCGTCTCCAT-3′) matches the core *att* site for several other SaPIs (e.g., SaPIm4, SaPImw2). Further comparison of the integrase sequence as well as the chromosomal integration site of the *tst*-carrying SaPI in MSSA 68111 with those from the previously characterized SaPIm4 and SaPImw2 showed 96% and 87% identity of intergrase genes, respectively, and the same chromosomal insertion site of all three, at 3′ end of *ssrP* (Figure 6B). In contrast, the organization of accessory genes in SaPI68111 including *sel*, *sec*, and *tst* genes, is most closely related to that of SaPIn1.

**Figure 7:**
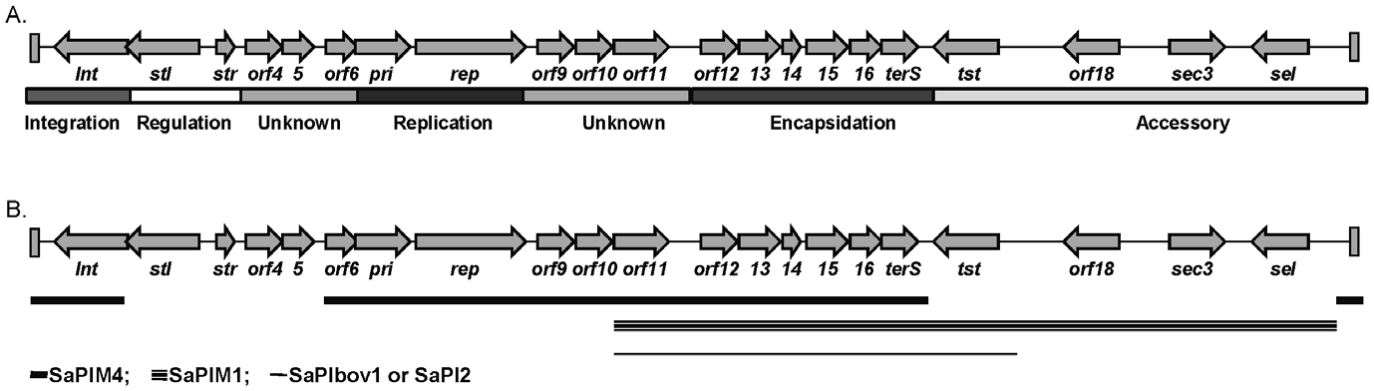
Illustrative representation of pathogencity island 68111 (SaPI68111). Arrows represent the location and orientation of open reading frames (OFRs), which were determined using the NCBI-GLIMMER program and by the relative position of ORFs within the island and/or similarity of the predicted gene products. (**A.**) An illustrative diagram of the SaPI68111 genome with different functional annotations presented at the bottom of the figure. (**B.**) A summary diagram of the homology regions between SaPI68111 and other close-related SaPIs. SaPIM4 (thick line), SaPIM1 (triple lines) and SaPIbov1 or SaPI2 (thin line).

**Figure 8:**
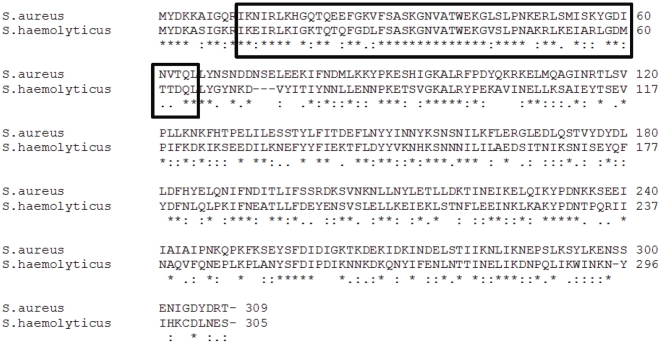
Amino acid alignment of predicted *stl* from MSSA68111 and hypothetical phage repressor protein SH2101 from Staphylococcus haemolyticus (Accession: YP_254016.1). The protein sequences were aligned by using the CLUSTAL alignment program. The Helix-turn-helix XRE-domain is highlighted in the black box. The asterisk denotes a position at which the two sequences have the same amino acid. Dots indicate the degree of homology when there is not complete sequence conservation.

Because of these findings, a complete genome sequencing of the *tst*-carrying SaPI in MSSA68111 was conducted using a PCR and bi-directional primer walking-based approach. In all, the 16,422-bp SaPI68111 (Accession: JN689383) was determined to have 21 ORFs potentially encoding proteins over 50 amino acids in length, 3 of which encoded staphylococcal exotoxins (*sel*, *sec*, and *tst)*, and many of which have homologs in SaPIM4, SaPIM1, SaPIbov1 and SaPI2 as indicated in Figure 7A, 7B and Table 4. These data suggest that the evolutional history of SaPI68111 probably includes at least one major recombination event with these SaPIs or other similar elements. Also interestingly, we found that the predicated product of *stl* in SaPl68111, a SaPI master repressor, shares 64% similarity (over 298 amino acids) to a hypothetical phage repressor protein of *Staphylococcus haemolyticus* bacteria (Accession: YP_254016; Figure 8). Particularly, the putative N-terminal HTH (helix-turn-helix) motif of SaPI68111-*stl* repressor belongs to the HTH -XRE-family of proteins and shows a high degree of 80% similarity (as much as 65% identity) to the HTH motifs of repressor protein of *Staphylococcus haemolyticus* but not to HTH motifs of most *S. aureus* (Figure 8). These data have led to the supposition that this gene was acquired by *S. aureus* through horizontal transfer from *Staphylococcus haemolyticus* during co-colonization of human skin [38].

**Table 4:** Relationships between SaPI68111 genes and those of other SaPIs.

SaPI68111		Mu50		RF122	RN3984	MW2	Annotation or Function
		SaPIM4	SaPIm1	SaPIbov1	SaPI2	SaPIMW2	
orf1	*int*	96(98)				87(100)	Integrase
orf2	*stl*						SaPI master repressor
orf3	*str*	88(91)				88(91)	Regulatory protein
orf4	HP			82(92)	83(91)		Hypothetical protein
orf5	HP	97(88)		95(88)	96(88)		Hypothetical protein
orf6	HP	96(100)	93(100)	96(100)	90(100)		Hypothetical protein
orf7	*pri*	96(100)	93(69)	93(69)	93(69)		Similar to DNA primase
orf8	*rep*	99(100)					Similar to replication initiation protein
orf9	HP	99(100)					Hypothetical protein
orf10	HP	99(100)					Hypothetical protein
orf11	HP	96(100)	96(100)	96(100)	95(100)		Hypothetical protein
orf12	HP	97(100)	97(100)	97(100)	96(100)		Hypothetical protein
orf13	cp	98(100)	98(100)	94(100)	98(100)		Capisid size determinant
orf14	cp	99(100)	99(100)	96(100)	100(100)		Capisid size determinant
orf15	cp	96(100)	96(100)	97(100)	95(100)		Capisid size determinant
orf16	HP	96(100)	96(100)	98(100)	96(100)		Hypothetical protein
orf17	*terS*	98(100)	98(100)	94(100)	97(100)	90(27)	Terminase small subunit
orf18	*tst*		100(100)	100(100)	100(100)		Toxic shock syndrome toxin-1
orf19	HP		100(100)			77(82)	Hypothetical protein
orf20	*sec3*		99(100)	95(100)		97(100)	Staphylococcal enterotoxin type C3
orf21	*sel*		100(100)	100(100)		99(100)	Staphylococcal enterotoxin L

***Note***: The similarities of SaPI68111 with other close-related SaPIs were determined with BLAST. Percentage of identity to corresponding gene in SaPI68111 and percentage of query coverage (in the bracket) were given in the table. Blank area indicated no corresponding genes.

### 4. Transcription of PVL and TSST-1

**Figure 9:**
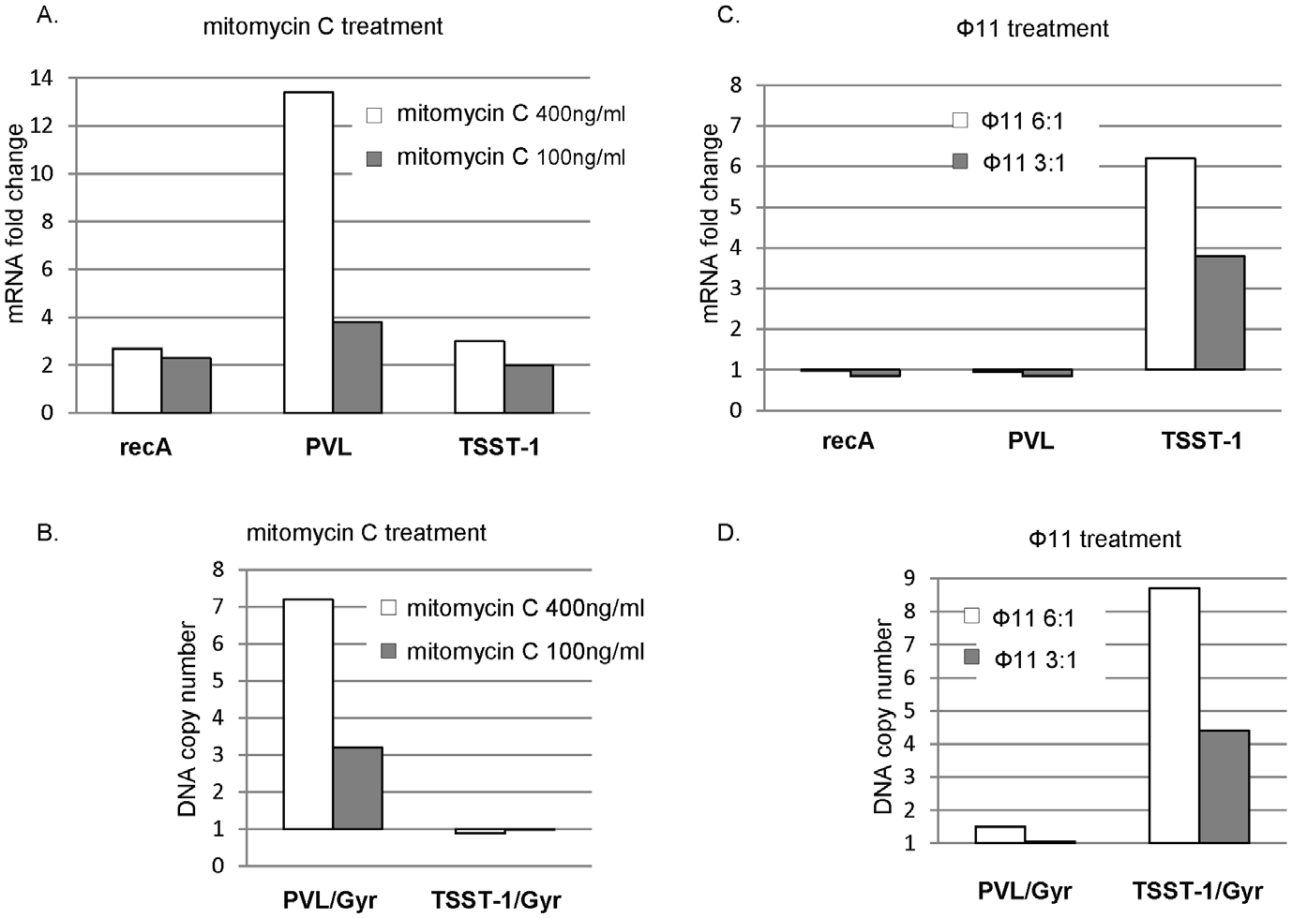
Comparative study of phage induction versus gene transcription in response to mitomycin C or phage 11 treatment. (**A. and B.**) Bacteria were treated with mitomycin C (400 ng/ml or 100 ng/ml) for 1 hr. (**C. and D.**) Freshly prepared phage 11 was added for 1 hr. One half of samples were used for RNA preparation followed by a quantitative RT-PCR analysis. Fold changes in gene expression of *rec*A, PVL and TSST-1 were normalized to the internal control gene gyrase (*Gyr*) and relative to no treatment control sample. The other half of samples were used for total genomic DNA preparation. Relative copy number of TSST-1 or PVL gene versus *Gyr* were calculated as described in *Materials and Methods*. Shown is one representative out of three independent experiments, all of which gave comparable results.

Increased PVL transcription after mitomycin C treatment has been attributed to an increase of phage copy number following phage excision and replication [39]. Consistent with this, our quantification of *lukS*-PV mRNA and DNA in MSSA68111 by real-time PCR confirmed that mitomycin C treatment resulted in a marked increases of both PVL transcript (Figure 9A) and PVL-DNA copy number (Figure 9B). Mitomycin C also activates *recA*-mediated autocleavage of phage repressors and resumption of the lytic cycle [40], [41]. Thus increased *recA* expression following DNA damage temporally coincides with increase phage-encoded gene transcription. Consistent with this notion, we observed a modest increase of *recA* expression in MSSA68111 following mitomycin C treatment (Figure 9A).

In contrast to PVL, mitomycin C treatment induced only a slight increase in TSST-1 mRNA and no excised form of *tst*-SaPI68111 was detected (Figures 9A, 9B). However, the abundance of both *tst* transcripts as well as the *tst* gene copy number were markedly and dose-dependently increased by helper phage 11 (Figures 9C, 9D). These results were observed in the absence of a *recA*/SOS response (Figure 9C). Similarly, PVL genes at both the DNA and mRNA levels also remained unchanged by phage 11 treatment (Figures 9C, 9D). Thus, *tst*-SaPI68111 induction (excision and replication) is positively correlated with TSST-1 transcription and does not involve the *recA*/SOS response.

## Discussion

Co-production of SEC, TSST-1 and PVL in MSSA68111 was proposed to contribute to the rapid demise of the young British patient with hemorrhagic pneumonia [9]. Further, the recent report of severe infections due to three separate clonal lineages of *S. aureus* co-producing TSST-1 and PVL [8] portends a possible emergence of hypervirulent *S. aureus* worldwide.

In addition to its epidemiologic association with fatal pneumonia, MSSA68111 is also of considerable interest from a purely genetic point of view. For unknown reasons, certain combinations of staphylococcal toxins are rarely present in the same clinical isolate. This is true for TSST-1 with staphylococcal enterotoxin B (SEB) and for TSST-1 with PVL. Thus MSSA68111 is a significant deviation from the norm. As such, it provides a unique opportunity to investigate the mechanisms responsible for this phenomenon and to gain insight into likely emergence and dissemination of the next *S. aureus* “superbug”.

We confirmed that MSSA68111 produced both TSST-1 and PVL. PVL production by MSSA68111 was comparable to other *S. aureus* clinical isolates we have tested [16], however the level of TSST-1 was low. The relatively low level of TSST-1 in MSSA68111 could not be explained by a non-functional *Agr/RNAIII* system since both alpha- and delta-hemolysins were produced. In addition, real-time RT-PCR showed that *RNAIII* transcripts in MSSA68111 were comparable to those from MRSA strains FPR3757 (USA300) and MW2 (USA400) (data not shown). Expression of *RNAIII* was inversely related to expression of *spa* gene in wild-type MSSA68111 but not in its isogenic RNAIII mutant (data not shown). The low levels of TSST-1 and PVL are also not an artifact of the ELISA-based detection system since their respective nucleotide sequences in MSSA68111 are highly similar to those of other strains (data not shown).

Although extensive studies have been conducted by others, the exact mechanism responsible for the wide variation in the amount of TSST-1 produced by different strains of *S.aureus*,is still unknown. A study of 152 TSST-1-positive MRSA isolates by Nagao et al showed that differences in TSST-1 production (up to 170-fold) is not directly correlated with the allelic variations of the *agr* [42]. In addition, their sequencing of the promoter region of the *tst* gene, the entire sigma factor B and *sar* loci revealed no relevant nucleotide changes among these strains [42]. Thus it is likely that the low level of TSST-1 in MSSA68111 may not simply be explained by one mechanism, but rather multiple known or unknown complex interconnected regulatory systems are operative. In addition, the low level of TSST-1 production in MSSA68111 could be a consequence of co-production of both TSST-1 and PVL toxins, as other investigators have shown that some toxins function as autorepressors or negative regulators of other exoproteins [43].

Bacteriophages mediate dissemination of genes and hence bacterial diversity. We show that the PVL-carrying phage from MSSA68111 (ФPVLv68111) is a variant of icosahedral-head type phage ФPVL. The basis for this conclusion is that ФPVL and ФPVLv68111 share similar phage structural genes, identical *XbaI* restriction profiles and phage integration sites, but different *SpeI* restriction fragment profiles. Among clinical isolates that have been studied, ФPVL is relatively rare in the United States but is more prevalent in Europe [28]. In contrast, the elongated-head type PVL-phage, represented by ФSa2usa, is currently dominant worldwide [44]. These findings suggest that ФPVL and ФPVLv68111 might have evolved from a common ancestor and that genetic drift may have occurred in one or both. Features unique to ФPVLv68111 may have permitted MSSA68111 to acquire the genes for TSST-1 production.

Phage-encoded toxin genes are not always transmissible often because integrated prophages are defective. However, ФPVLv68111 is fully mobilized as part of a *recA*-mediated SOS response and its induction results in enhanced *lukS*-PV transcription. These findings indicate functional ФPVLv 68111 can be readily transmitted to other *S. aureus* strains.

Staphylococcal pathogenicity islands (SaPIs) are other mobile genetic elements responsible for inter- as well as intra-genic spread of exotoxin genes. MSSA68111 carries a unique SaPI which we have designated SaPI68111. Like SaPIn1/m1, SaPI68111 carries a central region of sequence identity encompassing *sel-sec-tst* in the classical arrangement. However, unlike all known *tst*-carrying SaPIs, SaPI68111 is inserted in an *att* site close to the *ssrP* gene with a 15-bp repeat. Further, the integrase gene in SaPI68111 appears related to SaPImw2 and SaPIm4, but not to SaPIn1/m1 or any other known *tst*-carrying SaPIs. Most interestingly, the predicated *stl* gene in SaPI68111, a master regulator for SaPI EPR cycle, does not share any homology with any published sequence in *S. aureus*, but has 64% similarity with a hypothetical phage repressor of *Staphylococcus haemolyticus*. This unique combination of genetic characteristics suggests that SaPI68111 probably arose following at least one major recombination event with several SaPIs or other similar elements, whereas the regulatory gene (e.g., *stl* gene) might be acquired by *S. aureus* through horizontal transfer from other species (e.g., *Staphylococcus haemolyticus)* during co-colonization or infections.

Induction of SaPIs excision/replication requires specific helper phages, and in MSSA68111, chromosomal excision of *tst*-SaPI68111 was driven solely by helper phage 11. This finding is unique in that most SaPIs are specifically induced by phage 80α alone or in combination with phage 11. To our knowledge, none is induced solely by phage 11. Like PVL, excision/replication of *tst*-SaPI68111 was positively correlated with increased TSST-1 gene transcription. Whether features of SaPI68111 contributed to the emergence of PVL/TSST-1 co-producing *S. aureus* remains to be determined.

Several hypotheses exist to explain the observed specific paired toxin exclusion in *S. aureus*. First, exclusion results from competition for a single chromosome insertion site [45]. However, this cannot fully explain the mutual exclusion of TSST-1 and PVL in nearly all *S. aureus* since, as described above, the chromosomal location of PVL-carrying phages does not overlap with any currently reported *S. aureus* SaPIs including SaPI68111 and induction of *tst*-carrying SaPIs requires specific helper phages that are genetically distinct from PVL-encoding phages. Second, unknown “exclusion factors” encoded by the first genetic element block acquisition of the second genetic element [46]. If such factors exist and mediate toxin exclusion, it would imply that MSSA68111 is exclusion factor-deficient. Third, one mobile genetic element commandeers a key bacterial resource essential for transduction. Fourth, membrane depolarization after infection by the first element prevents secondary infection [47], [48]. Lastly, defects in the *Sau1* restriction modification system may permit multiple transduction events as has been suggested in *S. aureus* RN4220 [49]. Our genetic characterization of MSSA68111 offers new tools to investigate these possibilities.

In summary, our genetic analysis of a hypervirulent TSST-1/PVL co-producing CA-MSSA demonstrated that these potent virulence factors are each carried on unique and fully transmissible genetic elements. Together these findings portend a possible worldwide emergence of new hypervirulent *S. aureus*. Whether these elements are common among other recently described TSST-1/PVL co-producing *S. aureus,* and what their roles may be in overcoming mutual toxin exclusion, are under investigation.
